# Surgical repair of symptomatic medial clavicular fracture nonunion using a reversed distal clavicle plate: a report of 3 cases

**DOI:** 10.1016/j.xrrt.2023.05.011

**Published:** 2023-07-01

**Authors:** Christopher Anigwe, Nicholas Colyvas, Drew A. Lansdown

**Affiliations:** a University of California, San Francisco, School of Medicine, San Francisco, CA, USA; b Department of Orthopaedic Surgery, University of California, San Francisco, San Francisco, CA, USA

**Keywords:** Medial clavicle, Proximal clavicle, Fracture, Nonunion, Sternoclavicular joint, Reversed distal clavicle plate, Inverted distal clavicle plate, Patient reported outcomes

Fractures of the medial clavicle are incredibly rare, comprising only 2%-3% of all clavicle fractures.^13^ Patients are typically managed nonoperatively with good long-term outcomes.^18^ However, recent studies documented symptomatic nonunion rates approaching 8%-15% with conservative management.^4^^,^^6^^,^^14^

Surgical treatment of medial clavicle fractures (MCFs) is technically difficult given that they often result in a small medial fragment that remains reduced at the sternoclavicular (SC) joint, which limits potential options for achieving a stable construct to promote osseous healing. In addition, surgery also carries significant risk due to the intricate anatomy with nearby neurovascular and airway structures. Thus, it is associated with high complication and failure rates.^1^^,^^5^^,^^13^^,^^14^ Given how unusual these injuries are, the literature in this area is limited and the choice of surgical technique remains challenging as there are few described techniques. This has resulted in the utilization of different surgical approaches for symptomatic nonunion with varying outcomes, including stabilization with a palmaris longus graft, a dual plating technique, and multiple 2-staged approaches comprised of fixation and hardware removal.^1^^,^^2^^,^^4^^,^^16^

Fixation of the medial clavicle with a reversed or contralateral distal clavicle plate has produced good outcomes in cases without nonunion.^15^^,^^19^ Notably, Schultz et al^15^ described acute fixation of a MCF utilizing a contralateral distal clavicle plate across the SC joint. The purposes of this retrospective study are to describe a similar technique; however, using a reversed distal clavicle locking plate for fixation across the SC joint, and to also report the functional and pain outcomes of this surgery for patients with symptomatic nonunion of MCFs. We hypothesized that this technique would facilitate bony healing and improve pain and function in patients with medial clavicular fracture nonunion.

The Institutional Review Board approved this study. The patients were informed that data concerning their cases would be submitted for publication, and they all provided informed consent.

## Case reports

### Case 1

A 53-year-old man was evaluated at our patient office for 1.5 years of left shoulder pain after a fall down his stairs at home. He sustained fractures of his scapula, 9 ribs, cervical spine, and left clavicle with nonunion. He reported persistent left shoulder pain despite attempts at nonoperative treatment. He rated his pain as 7/10 on the Visual Analog Scale.

**Figure 1:**
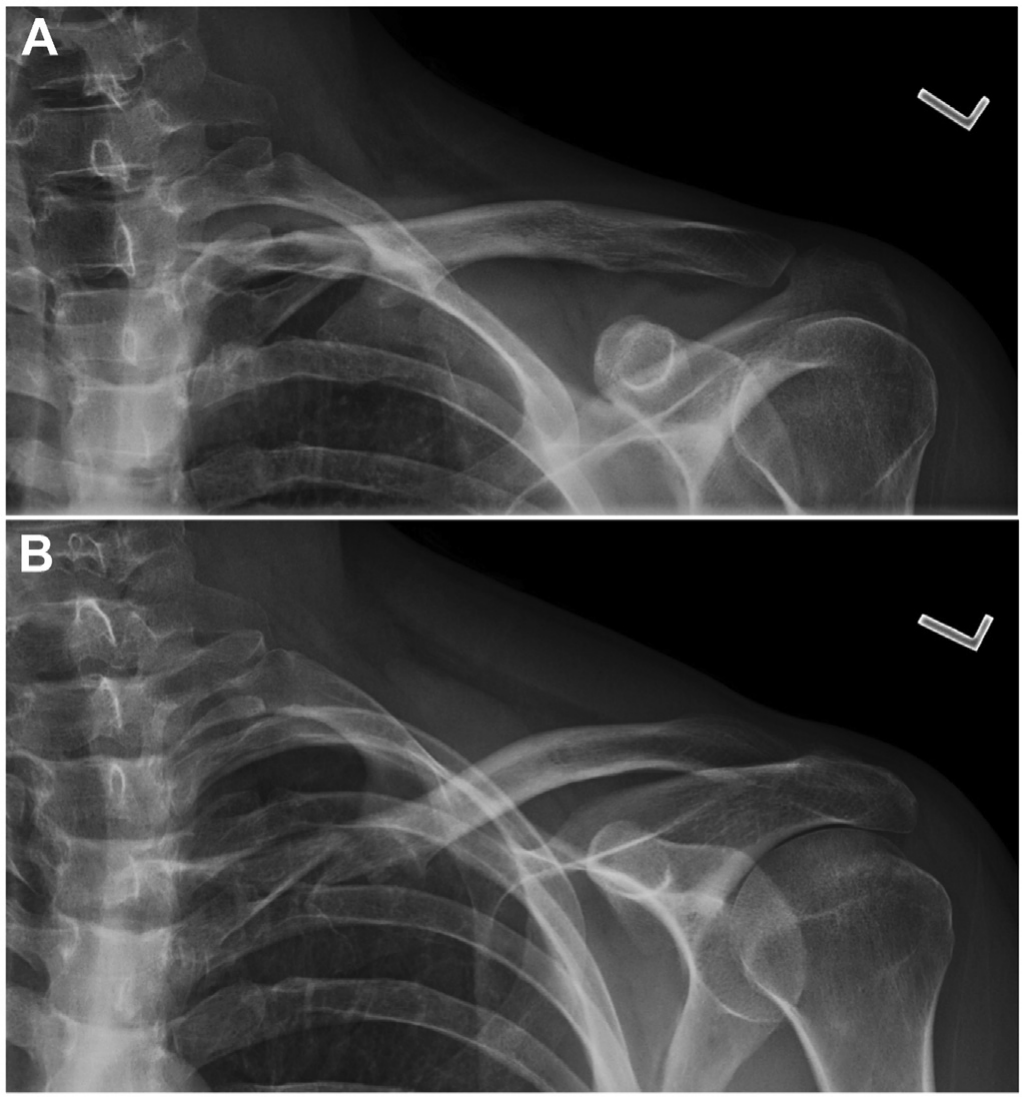
Two preoperative radiographic images, including anteroposterior view of the clavicle (**A**) and anteroposterior view with cephalic angulation (**B**) of the left clavicle demonstrate a nonunion of the medial clavicle, obtained at 10 months after initial injury.

Plain radiographs of the left clavicle were obtained and demonstrated the displaced fracture at the medial aspect of the clavicle (Fig. 1). Computed tomographic (CT) imaging revealed proximal clavicular nonunion with anterior and mild inferior displacement of the distal fracture fragment and chronic fragmentation at the fracture site. He was a smoker but quit prior to surgery. Four months after initial presentation, open reduction and internal fixation (ORIF) was performed on the affected clavicle (described below). Six-month postoperative CT imaging redemonstrated anatomic alignment and plate and screw fixation of the medial clavicle with bony callous and healing across the fracture site. The clavicle plate was removed 9 months after surgery.

He completed outcome surveys 3.7 years after the surgery (Table I). Patient-reported outcome measures, including the Quick-Disabilities of the Arm, Shoulder, and Hand,^7^ the American Shoulder and Elbow Surgeons shoulder score,^11^ and the Patient Reported Outcomes Measurement Information System- Upper Extremity,^9^ were collected. He was also surveyed on his satisfaction with the surgery on a 5-point Likert scale (1, Very Unsatisfied; 5, Very Satisfied), postoperative visual analog scale–pain score, and smoking history.

**Table I:** Demographics and postop outcomes of each patient.

	Case 1	Case 2	Case 3
Age at time of surgery, years	54	37	71
Smoking, pack-years	25	0	105
Time from fracture to surgery, years	1.8	1.1	1.4
Preop VAS pain score	7	8	4
Postop VAS pain score	1	2	6
Postop PROMIS-UE score	54.7	55.7	40.4
Postop QuickDASH score	2.3	13.6	27.5
Postop ASES shoulder score	88.3	81.7	45
Postop Satisfaction Rating	5	5	2

*VAS*, visual analog scale; *PROMIS-UE*, Patient Reported Outcomes Measurement Information System- Upper Extremity; *QuickDASH*, Quick-Disabilities of the Arm, Shoulder, and Hand; *ASES*, American Shoulder and Elbow Surgeons.

### Case 2

A 37-year-old man was evaluated for 10 months of left shoulder pain after suffering a left MCF from an off-road all-terrain vehicle crash in which he hit the handlebars and fell downhill. He was treated with nonsteroidal anti-inflammatory drugs and a sling that he wore for a month, though continued to have persistent left shoulder pain. He rated his pain as 8/10.

**Figure 2:**
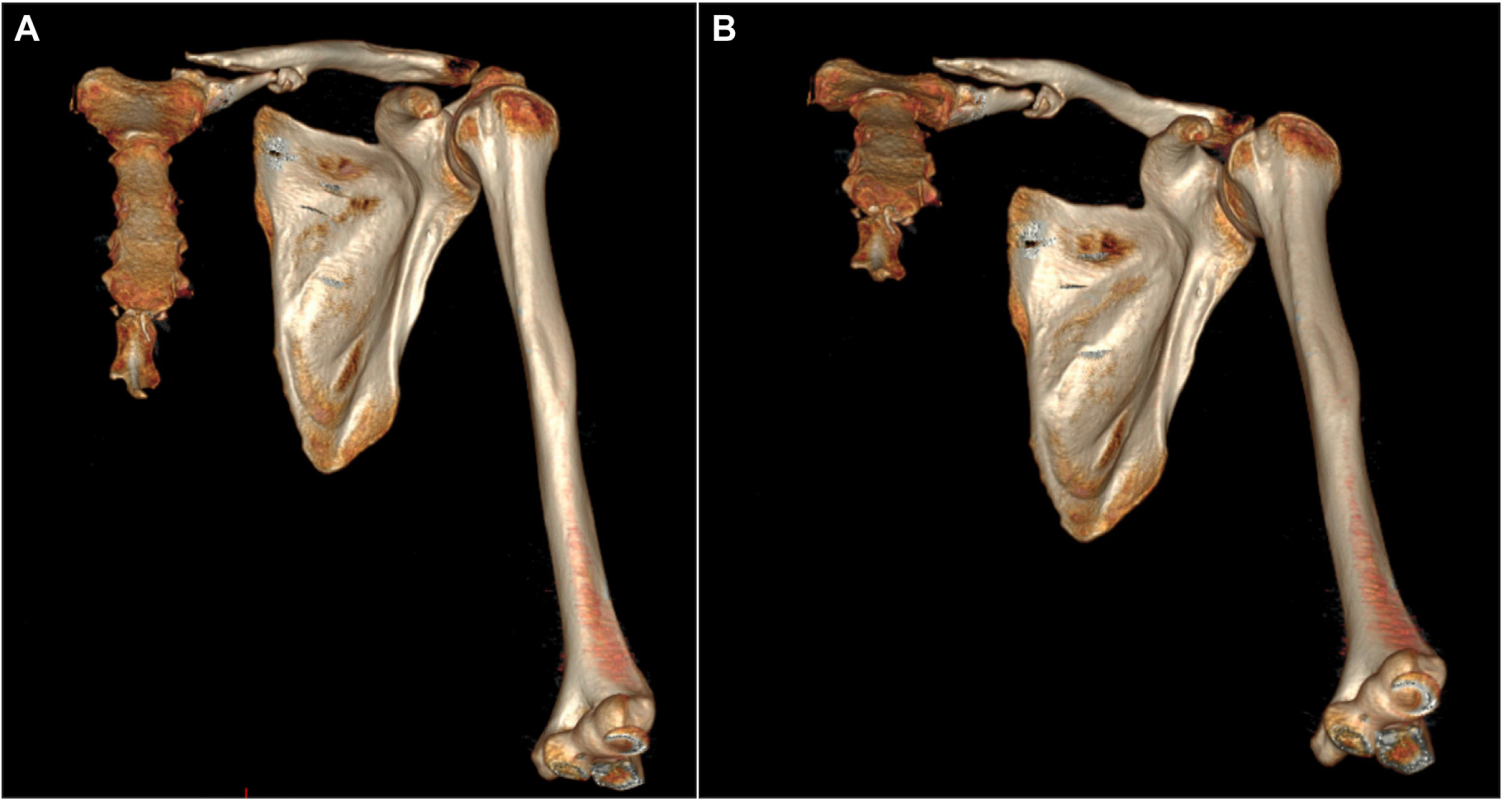
Preoperative three-dimensional reconstructions from computed tomographic imaging show a nonunion of the medial clavicle, obtained at 11 months after initial injury. Two perspectives are shown, including anteroposterior view (**A**) and anteroposterior with cephalic angulation (**B**). There is a short segment of medial bone, which remains reduced to the sternum at the sternoclavicular joint, and an oblique fracture pattern.

**Figure 3:**
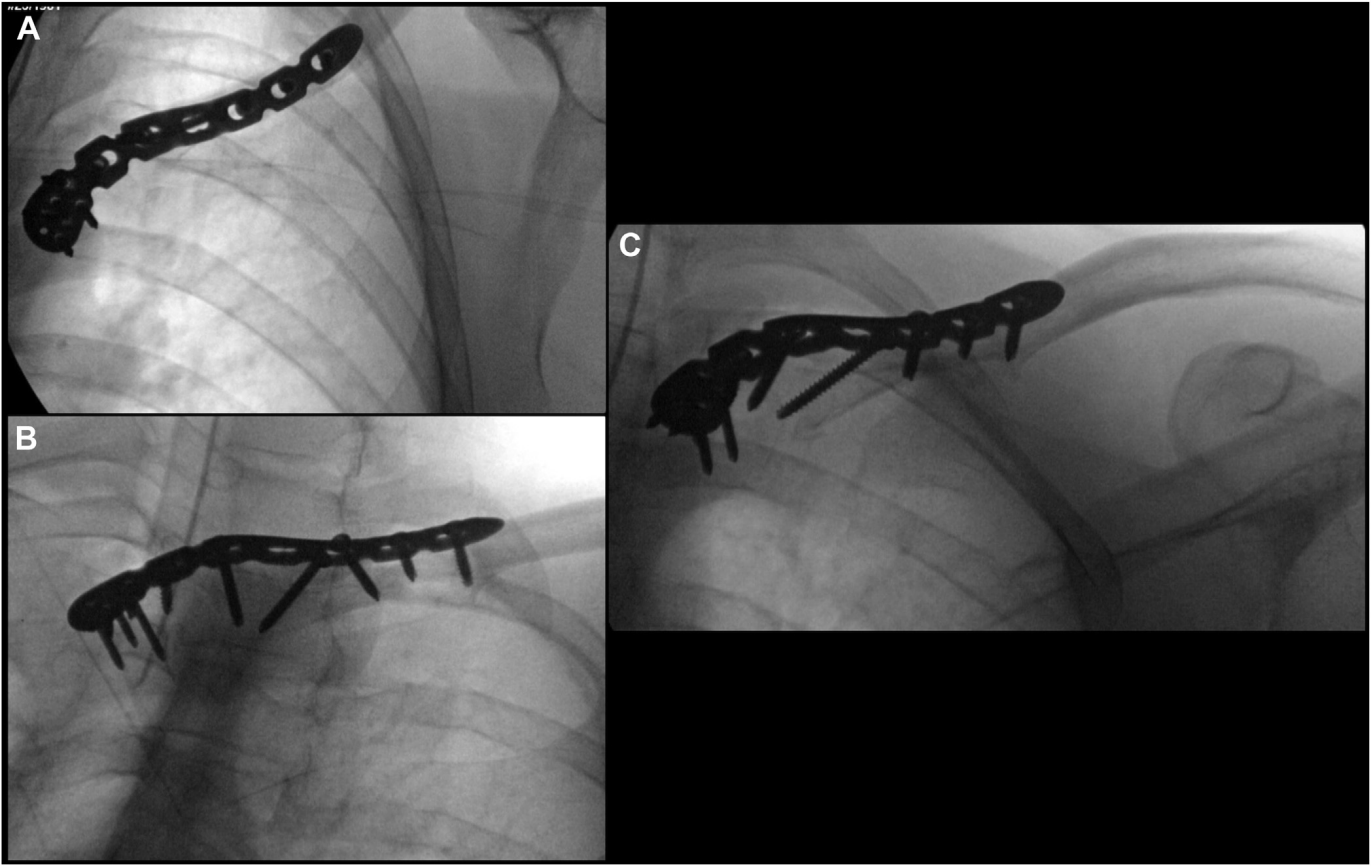
Intraoperative fluoroscopy images show fracture reduction and hardware placement, including the reversed distal clavicle plate and the interfragmentary lag screw. Views include (**A**) direct anteroposterior view of the clavicle plate positioned on the medial clavicle; (**B**) anteroposterior view of the clavicle; (**C**) anteroposterior view of the clavicle with cephalic angulation.

**Figure 4:**
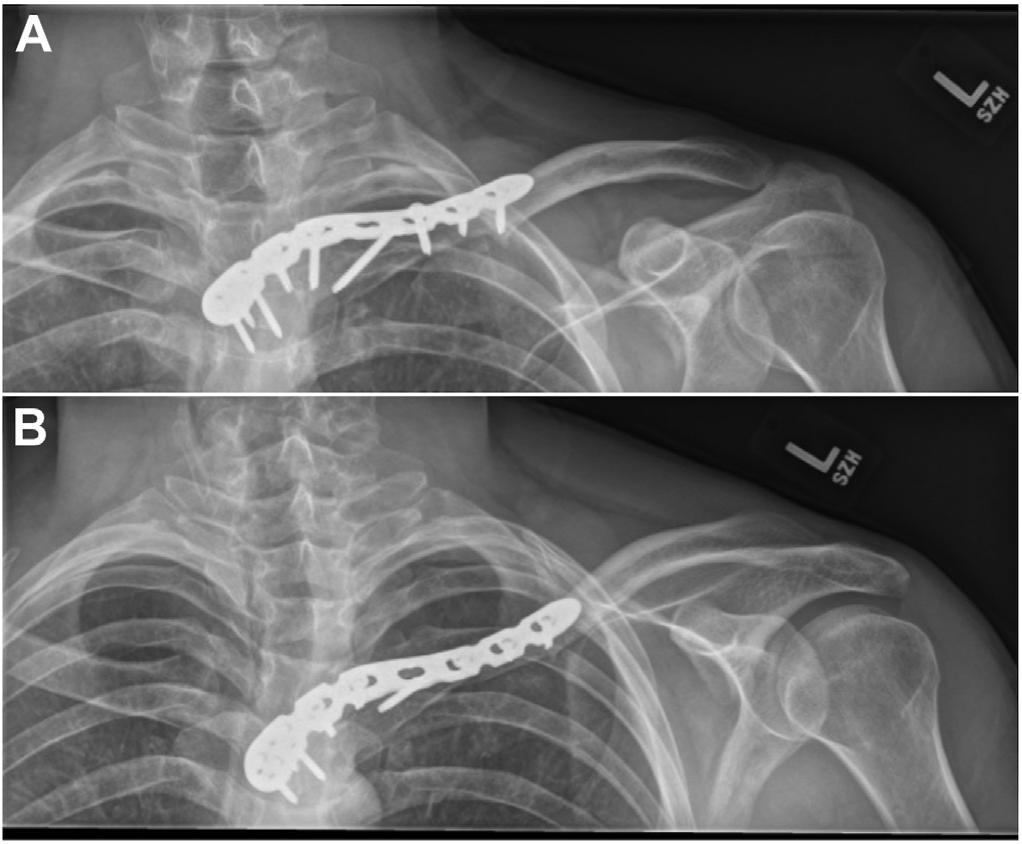
Postoperative radiographic imaging, including (**A**) anteroposterior view of the clavicle with cephalic angulation and (**B**) anteroposterior view of the left clavicle, shows fracture reduction and hardware positioning.

**Figure 5:**
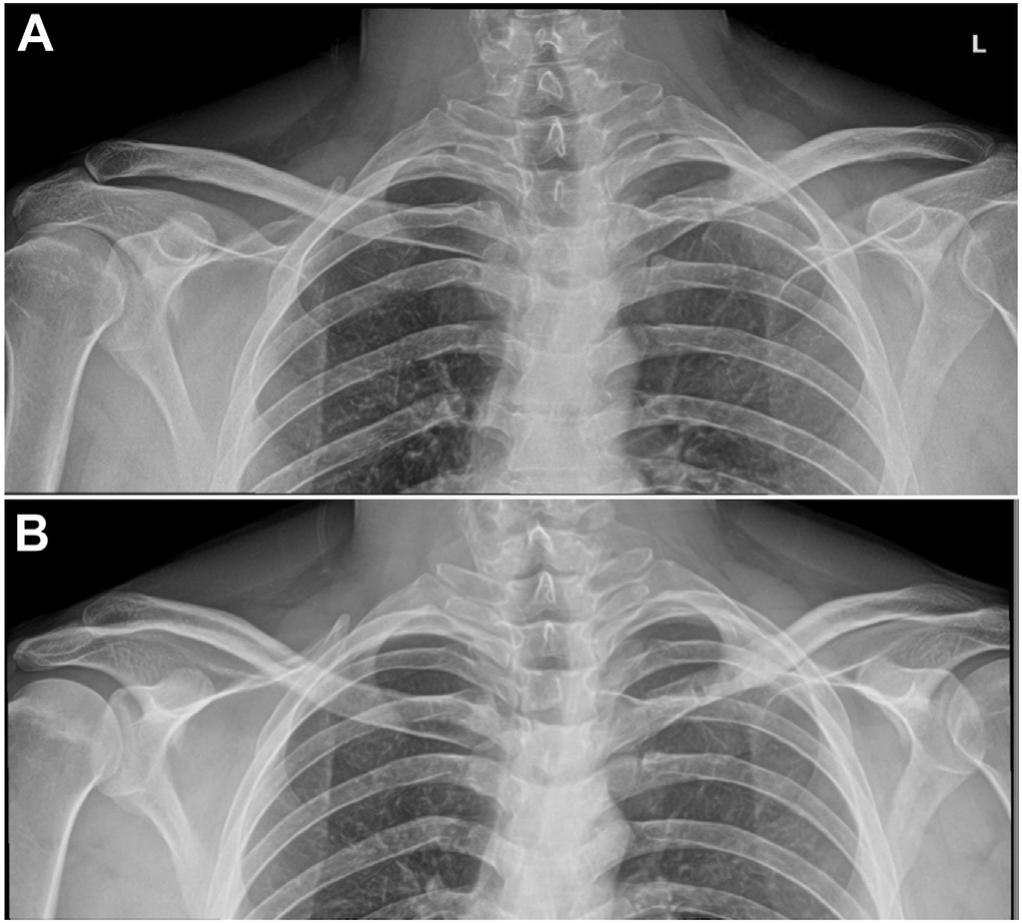
Two postoperative radiographic images, including (**A**) anteroposterior view of the clavicle with cephalic angulation and (**B**) anteroposterior view of the left clavicle, after hardware removal show a healed fracture.

**Figure 6:**
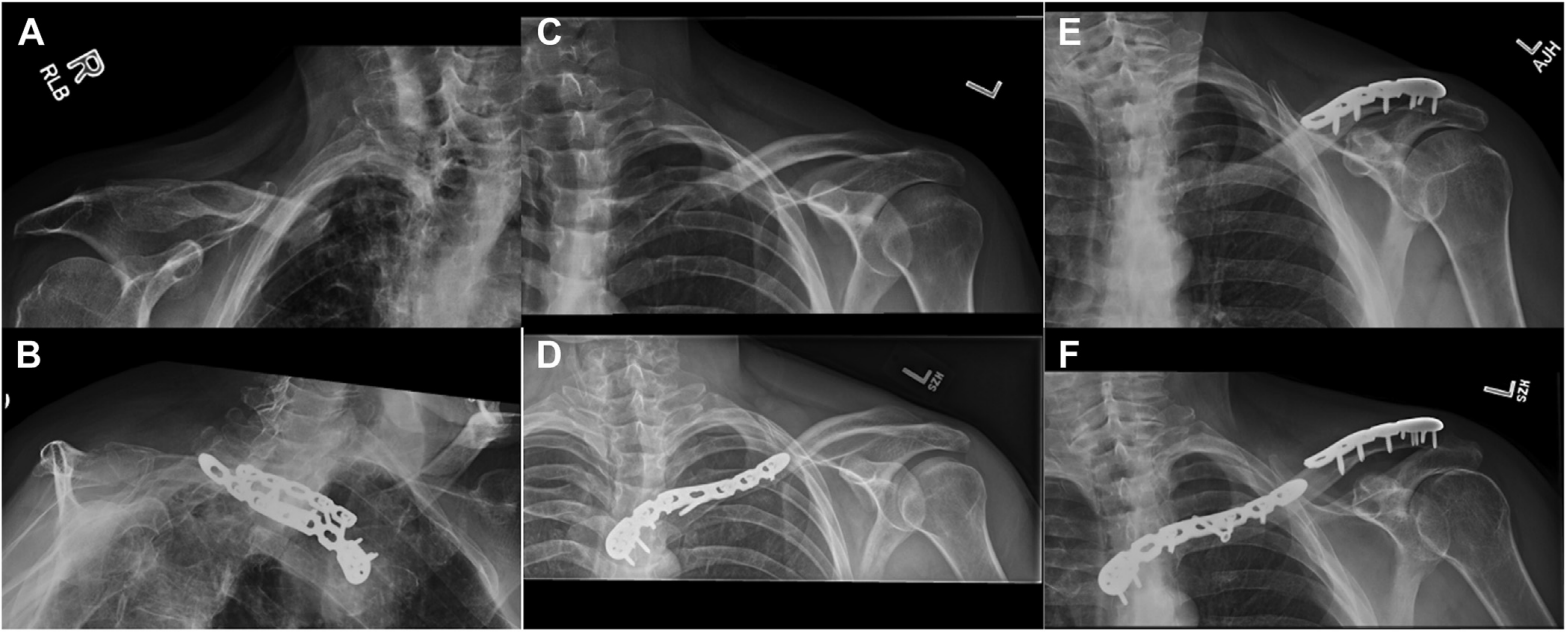
Preoperative and postoperative radiographs are shown for all three patients included in this report. Patient 1 is shown in (**A**) (preoperative) and (**B**) (postoperative). Patient 2 is shown in (**C**) (preoperative) and (**D**) (postoperative). Patient 3 is shown in (**E**) (preoperative) and (**F**) (postoperative).

A combination of radiographs and CT imaging demonstrated a displaced chronic fracture of the proximal to mid left clavicle (Figs. 2 and 3). 2 months after initial presentation, ORIF was performed on the affected clavicle (described below) (Figs. 4 and 5). This patient did not receive 6-month postoperative CT imaging; however, radiographic imaging that was done 10 months after surgery redemonstrated stable plate and screw fixation of the left medial clavicle and sternum in unchanged alignment. The hardware was removed 11 months after surgery (Fig. 6). He was surveyed 3.5 years after the surgery (Table I).

### Case 3

A 70-year-old man was evaluated for 8 months of right shoulder pain after a fall from a 10-foot platform at work in which he sustained fractures of his neck, ribs, and right clavicle. 2 days after his fall, he had an ORIF for the affected clavicle at an outside hospital. He continued to have pain and dysfunction in the shoulder and residual shoulder girdle deformity, likely due to clavicle shortening. He rated his pain as 4/10.

Combined radiograph and CT imaging demonstrated widening at the acromioclavicular joint and an oligotrophic nonunion of a fracture of the proximal clavicle with anterior-inferior displacement, both caused by the patient’s initial injury. Nine months after initial presentation, a revision ORIF was performed on the affected clavicle (described below). He was a previous smoker, but he self-reported that he quit smoking prior to the revision ORIF though no cotinine nicotine metabolite testing was performed. Six-month postoperative CT imaging revealed evidence of a persistent nonunion of the proximal clavicle with similar anterior-inferior displacement, and the plate lifting off the sternum. The findings correlated with increased plate prominence on physical exam and the patient noted that the plate was becoming irritating. He, thus, underwent hardware removal 13 months after surgery. At the time of hardware removal, he appeared to have a stable oligotrophic fibrous nonunion so further treatment was deferred. His survey results 3.3 years after surgery are included in Table I.

### Surgical technique

All procedures were performed by the 2 senior authors (DAL and NC). Under general endotracheal anesthesia, the patients were placed in a low beach chair position. A vascular surgeon was on standby for all procedures. A curvilinear incision was made from the mid clavicle to the mid sternum over the SC joint. The fracture ends were evaluated and cleared of fibrous tissue. A burr was used to decorticate the contacting surfaces. The fracture was reduced anatomically and held with reduction clamps. A lag screw was placed across the fragment when possible (Cases 1 and 2). A distal clavicle locking plate (DePuy Synthes, Raynham, MA, USA) was reversed 180 degrees (right plate for left shoulder) and placed with the cloverleaf locking paddle on the anterior surface of the sternum. Pilot holes were drilled unicortically, taking care to preserve the far cortex. Unicortical locking screws were placed to secure the plate to the sternum. Cortical nonlocking screws were then placed to secure the plate to the clavicle lateral to the fracture site. Intraoperative fluoroscopic imaging was utilized. If necessary, autograft bone graft was then packed around the nonunion site. For Case 3, a second reconstruction plate was placed superiorly at the clavicle to allow for improved stability.

We obtained postoperative CT scans in all patients immediately after surgery to evaluate hardware positioning. Patients were observed in the hospital setting overnight after surgery and discharged home the following day. Postoperative protocol consisted of a sling for shoulder immobilization for 8 weeks, treatment with a bone stimulator starting at 6 weeks postop, and physical therapy for gentle range of motion without manipulation of the shoulder beginning at 6 weeks postop.

Given the construct spanning the SC joint, we discussed hardware removal with all patients and planned for this at 1 year after primary fixation to restore shoulder motion. CT and/or radiographic imaging was performed at or after 6-months postop to confirm union to move forward with eventual hardware removal at 1-year postop. The fracture site was inspected at the time of hardware removal. Motion across this site was checked after the implant was removed.

## Discussion

Fixation of MCF nonunion with a reversed distal clavicle locking plate spanning the SC joint allowed for stable fixation of the nonunion site, improvement in pain, and good postoperative functional status of the affected shoulder for 2 out of our 3 patients. MCFs represent both a rare injury and a technically challenging problem to manage successfully. Various surgical approaches are described in the literature; however, the optimal approaches are not yet determined. Some authors suggested wire fixation, T-plate fixation, or advice for or against plating across the SC joint, all with varying outcomes.^2^^,^^3^^,^^5^^,^^13^^,^^19^ Some noted drawbacks of plating across the SC joint are that the plate is subject to high torsion forces during shoulder motion and that another procedure is required to remove the hardware and restore motion.^2^^,^^13^^,^^19^ However, locking plate fixation has produced good results when fixation over the sternum is a necessity, especially when compared to utilization of a nonlocking system.^13^ As described here, fixation across the SC joint with a reversed distal clavicle locking plate can be considered as a possible option when medial fracture fragment size precludes potential fixation in this area.

Fixation of the medial clavicle with a reversed or contralateral distal clavicle plate has been previously described in a few other studies. Wang et al^19^ reported a case of a 40-year-old man with a displaced MCF. In this case, fixation was limited to the medial clavicle and the authors reported rigid fixation, the lack of a plate removal due to less soft tissue irritation, and avoidance of damage to nearby structures given ability for placement of the reversed distal clavicle plate on the superior aspect of the medial clavicle. Schultz et al^15^ also described a case of a displaced MCF; however, achieved adequate fixation with a contralateral distal clavicle plate across the SC joint. They found that the precontoured plate designed for the contralateral distal clavicle closely resembled the anatomy of the medial clavicle and SC joint and allowed for ease of placement. However, plate removal was ultimately required after healing due to restricted movement across the SC joint. In contrast to our study where we included multiple cases of patients who were all treated for established nonunion and each had more than 3 years of follow-up, both case studies described procedures which were done for primary treatment, featured only 1 patient, and had 1 year and 8-month follow-up, respectively.

The approach for fixation of a MCF is technically demanding since the fracture pattern often consists of a short segment of medial bone, which presents challenges for fixation. This prompted us to span across the SC joint. Utilizing unicortical locking screws may allow for safer instrumentation in this region while also limiting the need for fixation at the small segment of bone at the medial clavicle. While intraoperative fluoroscopy was utilized to evaluate reduction and hardware placement, we also obtained postoperative CT scans to fully evaluate screw length with plans to change screws if any were too long. While no patient required immediate reoperation, we believe this is an important step to ensure appropriate hardware placement in this region. Alternatively, surgeons could consider intraoperative O-arm imaging to immediately evaluate screw length.

Case 3 was unsatisfied with his surgery and reported persistent pain and poor functional status of his shoulder at the time of hardware removal, and his postoperative scans demonstrated a persistent nonunion at 6 months. Notably, this patient was unique considering he had a prior attempted surgery of the injured clavicle which failed to improve his pain. Not only was the initial ORIF not successful at healing his nonunion, but the patient also had persistent acromioclavicular joint widening and shoulder girdle deformity by the time he presented to us 8 months after his initial injury. The concurrent injuries that occurred prior to our revision ORIF likely contributed to the patient’s poor outcome. In addition to the prior surgery, he was also the only patient in whom a lag screw could not be placed at the fracture site, which may have complicated our technique and contributed to the eventual hardware failure.

Case 3 had a prior extensive smoking history of 105 pack-years. Cotinine nicotine metabolite testing was not performed to confirm discontinued nicotine usage prior to the revision ORIF. Studies have demonstrated the harmful effects of smoking on bone healing and the elevated risk of progression to nonunion in midshaft clavicle fractures.^10^^,^^12^ Furthermore, the dose-dependent effect of smoking on fracture healing may explain the difference in outcomes compared to our patient with a 25 pack-year smoking history.^17^ Infection was not ruled out with tissue cultures at the time of his revision surgery, although it is one of the known risk factors for progression to nonunion.^8^ Although the cause of this patient’s poor outcome is likely multifactorial, it suggests that an alternative approach may be necessary to repair medial clavicle nonunion in patients with a previous attempted surgery or who are at high-risk of persistent nonunion.

The rarity of MCFs coupled with the scarce literature on this injury make it challenging to draw any generalizable conclusions. Future research would benefit from collecting preoperative patient reported outcome survey data to evaluate the impact of this procedure on shoulder function over time and provide a more comprehensive assessment.

## Conclusions

Internal fixation of the medial clavicle with fixation across the SC joint with a reversed distal clavicle plate is an option for patients with symptomatic medial clavicle nonunions.

## Disclaimers:

Funding: No funding was disclosed by the authors.

Conflicts of interest: The authors, their immediate families, and any research foundation with which they are affiliated have not received any financial payments or other benefits from any commercial entity related to the subject of this article.

Patient consent: Obtained.
